# Approach to the liver transplant early postoperative period: an institutional standpoint

**DOI:** 10.5935/0103-507X.20190076

**Published:** 2019

**Authors:** Beatriz Amaral, Madalena Vicente, Carla Sofia Maravilha Pereira, Teresa Araújo, Ana Ribeiro, Rui Pereira, Rui Perdigoto, Paulo Marcelino

**Affiliations:** 1 Unidade de Terapia Intensiva, Hospital Curry Cabral - Lisboa, Portugal.; 2 Departamento de Imunoterapia, Hospital Curry Cabral - Lisboa, Portugal.; 3 Unidade de Transplante Hepático, Hospital Curry Cabral - Lisboa, Portugal.

**Keywords:** Liver transplantation, Postoperative period, Intensive care, Perioperative period/adverse effects, Liver/surgery

## Abstract

The liver transplant program in our center started in 1992, and post-liver transplant patients are still admitted to the intensive care unit. For the intensive care physician, a learning curve started then, skills were acquired, and a specific practice was established. Throughout this time, several concepts changed, improving the care of these patients. The practical approach varies between liver transplant centers, according to local specificities. Hence, we wanted to present our routine practice to stimulate the debate between dedicated teams, which can allow the introduction of new ideas and potentially improve each local standard of care.

## INTRODUCTION

Liver transplant (LT) patient care consists of specific approaches, different from the usual major abdominal surgery approaches. In the literature, there are general descriptions regarding the postoperative care of surgical patients, yet a description of a practical approach to LT is deemed necessary. Over the years, several concepts have changed. Practices vary between centers and depend on specific acquired experience and local characteristics. Hence, we thought it would be useful to present an approach developed in our transplantation center through the years since the beginning of our LT program in 1992. Only adult patients are admitted, and most organs are from deceased donors, with the particular exception of domino transplant, which uses livers from familial amyloidosis polyneuropathy patients.^(1-3)^

The surveillance of the patients submitted to LT is performed by a multidisciplinary team,^(2,4,5)^ with different specialties and consequently distinct skills and functions. We present the intensive care perspective of the first 48 hours after LT. Specific surgical complications or techniques and immunosuppression issues for long-term follow-up are beyond our scope.

### General considerations

The events observed in the first hours after LT are mainly conditioned by intraoperative instability, graft characteristics, and recipient pre-LT clinical status (Table 1).^(4)^ Important information for the intensive care unit (ICU) is the quantity of transfusion, the quantity of administered normal saline fluid, the need for vasopressors, urine output, general hemodynamic characteristics and other intraoperative complications.^(4)^ Intraoperatively, LT is characterized by three stages: hepatectomy phase, anhepatic phase and reperfusion phase.^(6)^ The last is critical and of most importance due to a rise in right ventricular and intracranial pressures, arrhythmias and potassium load, cytokine load, emboli and worsening of coagulopathy.^(4)^

**Table 1:** Immediate complications after liver transplant

Early liver graft dysfunction
Primary dysfunction/malfunction of the graft
Early rejection
- Acute cellular rejection
- Absence of immunosuppression
Nonspecific cholestatic syndrome
Drug hepatotoxicity
Surgical technique complications
Arterial complications
- Hepatic artery thrombosis
Portal vein thrombosis
Hepatic venous obstruction
Biliary complications
- Bile leak or fistula
- Biliary stricture
Medical complications
Blood loss and acute hemorrhage
Hemodynamic complications
Acute renal failure and altered electrolytes
Respiratory dysfunction
- Hypoxemia and hepatopulmonary syndrome
Altered neurologic status
Infections
- Donor organ
- Transfused blood products
- Reactivation of previous infection
- Exogenous microorganisms and endogenous flora

Source: Adapted from Moreno R, Berenguer M. Post-liver transplantation medical complications. Ann Hepatol. 2006;5(2):77-85.^(7)^

The intensive care physician should also be alert to previous disease status before transplant, evaluated by clinical scores, such as Model for End-Stage Liver Disease (MELD) and Child-Pugh.^(5)^ In our LT center, in the first 2000 liver transplants, the mean MELD score was 19.5 ± 7.3.

A new clinical entity was recently identified, acute-on-chronic liver failure (AoCLF).^(6)^ The results of LT in this situation and in most ill patients with 3 or more failing organs is still a matter of controversy; data are still scarce, but the mean and long-term survival after LT are usually poor.

At admission, a full laboratorial evaluation is needed, and re-evaluation is usually performed 6 to 12 hours after admission in a stable patient; however, in those unstable, it should be repeated as frequently as needed.^(2,4)^

It is important to emphasize that ICU specialists should be in close contact with other team members, namely, surgeons, hepatologists, hemotherapists and imaging specialists.^(4)^ If any event occurs in the ICU, the other team members must be promptly informed.

### Cardiovascular system and fluid therapy

Hypotension is probably the most common clinical complication in the early postoperative period and should be actively prevented and aggressively managed. Graft ischemia occurs during hypotensive status, compromising the graft function recovery.^(4,7,8)^ The hemodynamic performance of cirrhotic patients is characterized by high cardiac output and low peripheral vascular resistance.^(9,10)^ Multiple arteriovenous shunts end in this hyperdynamic circulation and redistribute the body fluids, which can result in a relative central hypovolemia.^(8,10)^ This pattern closely resembles the hemodynamic pattern of sepsis and other inflammatory conditions that occur without infection. Most hepatic complications and liver cirrhosis decompensation are accompanied by systemic inflammation. In turn, this previous hemodynamic pattern limits the adaptive response of these patients to inflammatory (infectious and noninfectious) conditions, leading to sudden and severe hypotension and impaired organ perfusion.^(10)^ Situations such as acute anemia and ischemia-reperfusion injury (IRI), as well as the surgical procedure itself, are the main factors responsible for complications.^(8)^ Thus, a distributive or vasogenic pattern of hypotension (high cardiac output and low peripheral vascular resistance) is expected. When interpreting hypotension in these patients, conditions such as hypovolemia, blood loss and low peripheral vascular resistance due to the inflammatory state must be assessed. A method for hemodynamic evaluation, invasive or noninvasive, should be used to establish the physiological pattern and support any therapeutic actions. No specific method is considered superior, but generally invasive hemodynamic monitoring (PiCCO^®^ and Swan-Ganz Pulmonary Artery Catheter) might be considered.^(10)^

Fluid management is essential to provide euvolemia and obviate hypotension while simultaneously avoiding high volume loading.^(11,12)^

One should be aware that the postoperative period of LT is an inflammatory condition characterized by increased capillary permeability and consequent fluid redistribution to the interstitial space.^(8)^

Hemodynamic parameters of fluid responsiveness are not without pitfalls. Either static (central venous pressure, or pulmonary capillary wedge pressure) or dynamic (only validated in those mechanically ventilated without respiratory stimuli) hemodynamic parameters of fluid responsiveness are erratic but the only available tools for therapeutic guidance. When possible and if greater than 15%, the systolic volume variation in patients in sinus rhythm signifies that the goal of fluid administration will most likely be achieved. In our practice, rapid administration of 100 - 200mL of normal saline is effective, and if the patient remains hypotensive, a continuous vasopressor infusion should be initiated to avoid further large volume infusion and long periods of hypotension.^(11,12)^ This strategy is also valid in the presence of bleeding. Large amounts of fluids are deleterious and responsible for hemodilution, dilution (especially patients with low urine output) of circulating clotting products and destruction of already formed small clots.^(13)^

Although not routinely used in our center, some situations may require volume expansion with albumin solutions. This is particularly the case for continuing ascites drainage after LT, which can last for days and is managed with albumin support.

A normal hemodynamic recovery and lowering serum lactate are desirable; a return of sequestered liquid is expected, and a central venous pressure not greater than 5cmH_2_O ensures a pressure gradient between the portal and central circulation, resulting in better graft perfusion.^(4,13)^ It has been suggested that graft function may be affected by plasma glucose level.^(4,13)^ If hypoglycemia occurs in LT patients, it should be immediately managed with boluses of 30% or 50% dextrose and continuous infusions of 5% or 10% dextrose.

Cirrhotic cardiomyopathy is a distinct entity that is recognized earlier in patients with cirrhosis and no alcohol consumption.^(14)^ It is characterized by both systolic and diastolic alterations and reduced membrane activation to adrenergic stimuli. It can be a cause of unrecognized heart failure and lead to electrical instability after LT, which can interfere with the outcome.

### Ischemia-reperfusion injury

Liver ischemia during LT is unavoidable. This interruption in blood flow as well as the surgical trauma leads to multifactorial cell dysfunction and inflammatory mediator release, which are further exacerbated during the revascularization/reperfusion phase due to oxidative stress.

This clinical phenomenon is known as postreperfusion syndrome (PRS) or is perhaps more accurately defined as IRI.^(15,16)^

During LT surgery, both surgical trauma and IRI have the potential to produce inflammatory reactions that can influence long-term liver graft function.^(17)^ Almost all patients experience some degree of IRI, and 3 distinctive periods must be considered to potentially contribute to liver damage.^(17)^ The first is after explantation of the liver from the donor, when the liver is perfused with preservation solutions and stored in ice for transport to the transplantation center (cold ischemia period). After that, a short period of rewarming follows the performance of the anastomosis (warm ischemia period). Finally, once the hepatic artery anastomosis is finished, the liver is fully vascularized, and the graft temperature increases to 37ºC, starting the period of reperfusion.

From the immunological standpoint, in the reperfusion phase, two distinctive periods can be singled out: generation of reactive oxygen and nitrogen species characterizes the first, in the initial 1 - 2 hours of after reperfusion, leading to an initial stage of the inflammatory process and oxidative stress. The nitric oxide produced during this period is linked to mitochondrial damage and dysfunction. The second period, occurring 6 - 48 hours later, is characterized by cellular injury and activation of immunocompetent cells, such as Kupfer cells and neutrophils.^(16,17)^

Cardiovascular dysfunction in IRI is characterized by a decrease in mean arterial blood pressure, heart rate and systemic vascular resistances, usually associated with an increase in pulmonary vascular resistance index and pulmonary arterial pressure, reflecting pulmonary vasoconstriction.^(15)^

This hemodynamic pattern is attributed to graft-induced temperature, pH and electrolytic changes as well as inflammatory mediators, impaired right ventricle function, cardiac preload variations during LT surgery or perioperatively and pre-existing cirrhotic cardiomyopathy.

It is of the utmost importance to maintain adequate hemodynamic stability in the perioperative setting to restore graft liver function and to safely recover the surgical patient in intensive care.^(16)^

### Acute hemorrhage and coagulopathy monitoring

Diagnosis of postoperative bleeding is clinical and laboratorial.^(1,4,7,18)^ Clinical signs such as tachycardia, hypotension and blood loss from the abdominal drains raise suspicion, which is confirmed by a decrease in hemoglobin levels.

Early postoperative bleeding is defined as any bleeding requiring more than 3 units of red blood cells within 12 hours or surgical reintervention.^(18)^ Causes may include poor graft function, dilutional coagulopathy, hypocalcemia, hypothermia, acidosis, hyperfibrinolysis and surgical issues.^(18)^

When evaluating blood losses, two important conditions should be differentiated (Table 2): bleeding diathesis and a bleeding vessel due to incomplete intraoperative hemostasis.^(18,19)^ These conditions are managed differently: bleeding diathesis is supported medically and with clotting products (surgery rarely needed); the treatment of a bleeding vessel is usually surgical (Table 2).

**Table 2:** Bleeding due to coagulation abnormalities versus vascular abnormalities

	Bleeding diathesis	Bleeding vessel
Timeline characteristic	Usually slower, within hours	Usually rapid, within minutes
Timeline after transplant	Early	Usually 24 hours
Drainage fluid hematocrit	Usually < 50% blood hematocrit	Usually > 50% blood hematocrit
Blood pressure	Hypotension is established progressively	Usually a rapid decrease is observed

In the management of postoperative coagulopathy, the risk of bleeding should be counterbalanced by the risk of thrombosis, mainly hepatic artery thrombosis (HAT).^(1,4,20,21)^

There are no validated guidelines for the threshold values of hemoglobin, platelets, prothrombin time (PT), partial thromboplastin time (apTT) or thrombin time (TT) that will determine the need for transfusion of blood products, and clinical protocols vary between centers.^(20,21)^ However, there is clinical evidence of an association of blood product transfusions with increased patient morbimortality, increased rates of acute transfusion-related lung injury (TRALI) and infections.^(22)^

In our center, we use the classical coagulation tests - PT, apTT, TT, d-dimers (DD) and factor V, which reflect the procoagulant pathway - and on the first day after LT, we also monitor natural anticoagulants (antithrombin III, protein C and S), as they recover slower than the procoagulants, leading to a transient prothrombotic state.^(23-26)^

The expected perioperative level of hemoglobin is approximately 7 - 9g/dL, depending on the patient's clinical condition: 7g/dL for those with no risk factors and up to 9g/dL if there are previous conditions such as ischemic heart disease or brain injury.^(5)^ The baseline value for platelet transfusion is 20 x 10^9^/L, with the exception of patients in active bleeding, to whom the platelet count must be at least 50 x 10^9^/L.^(5,7,18)^ The aim is to restrict blood product use as much as possible.

There is no need for fresh-frozen plasma to correct moderate International Normalized Ratio (INR) elevations (INR < 1.8) unless there is active bleeding or anticipated surgical reintervention.^(20)^ However, for volume depletion in the initial post-LT hours, it is advisable, when possible, to use fresh-frozen plasma instead of crystalloids (to avoid dilutional coagulopathy^(11,12)^) and colloids (which interfere with coagulation and platelet function). If there is a risk of volume overload, vitamin K-dependent coagulation factor deficits are managed by administering prothrombin complex concentrate.^(25)^

Fibrinogen levels must be taken into account in case of active bleeding or before invasive procedures to keep them between 1.5 and 2.0g/L.^(1)^ This can be achieved with the infusion of fibrinogen concentrate and/or cryoprecipitate, especially in the uremic patient, as it also contains von Willebrand factor.^(20,26)^

In the early postoperative period, microvascular hemorrhage can be a sign of hyperfibrinolysis. Rotational thromboelastometry (ROTEM^®^) is a point-of-care method enabling rapid and sensitive evaluation of fibrinolysis.^(21,25,26)^ In uncontrolled hyperfibrinolysis with clinical repercussion, antifibrinolytic agents such as tranexamic acid should be used.^(18,26)^ Despite their safety, with no evidence of increased thromboembolic complications, they are only indicated in active bleeding (and not as a prophylactic measure) and should be avoided in patients with high known thrombotic risk (Budd-Chiari syndrome, thromboembolic diseases).^(18,26)^

In most cases, the administration of blood products is sufficient to compensate for moderate postoperative hemorrhage. However, in the presence of severe coagulation changes with uncontrollable bleeding, even massive infusion of blood products and antifibrinolytics drugs may be ineffective.^(21,25,26)^ In this setting, recombinant activated factor VIIa (rFVIIa) has been shown to be effective; its prophylactic use is not recommended, as it increases the risk of HAT.^(27)^

### Respiratory system

Early extubation in appropriate patients has been shown to improve graft function, reduce ICU stay and decrease the nosocomial infection rate.^(28)^

Pleural effusion is characterized by single-sided (generally right) transudate resulting from abdominal ascites transferred through diaphragmatic defects and impairment of lymphatic drainage due to surgery. Pleural effusion may increase in the first postoperative week, but it usually resolves within the following weeks without any intervention. The causes of postoperative atelectasis include pleural effusion, right diaphragm paralysis, bronchial obstruction, prolonged immobilization, insufficient inspiration due to pain and impaired clearance of secretions.^(5)^ Right diaphragm paralysis can occur when the right phrenic nerve is injured during surgery, culminating in atelectasis of the right lower lobe.^(28,29)^

Acute respiratory distress syndrome (ARDS) is one of the most severe respiratory problems after LT. Severe reperfusion syndrome, massive transfusion (TRALI),^(29)^ long operation time and infection are major causes of ARDS.^(29)^ Management of respiratory complications, namely ARDS, primarily involves supportive treatment (antibiotics, oxygen therapy, prevention of hypervolemia, drainage of massive pleural effusion and ascites, bronchoscopic aspiration). However, if there are signs of respiratory failure, mechanical ventilation support should be reinitiated without delay.^(7,29)^

Another cause of hypoxemia after LT is hepatopulmonary syndrome (HPS), defined as a defect in arterial oxygenation induced by pulmonary vascular dilatation in the setting of liver disease.^(30)^ This syndrome is present in 10 - 32% of patients with cirrhosis and occasionally unnoticed pre-LT; however, it may have its inaugural presentation within the first 24 hours postsurgery and should be diagnosed by a bedside echocardiogram.^(30)^ It is thought to be related to postoperative pulmonary vasoconstriction resulting from an abrupt change in the vascular mediators entering the lung from the hepatic effluent. Due to possible remodeling and impaired vasoconstriction in dilated HPS vessels, nondilated (normal) pulmonary vessels may vasoconstrict disproportionately, resulting in further increases in flow through dilated HPS vessels and, consequently, a transient worsening in the underlying diffusion-perfusion (VQ) mismatch of HPS, culminating in severe hypoxemia.^(30)^

Another cause of hypoxemia is portopulmonary hypertension. This situation affects 2 - 5% of liver cirrhosis patients, although it has severe clinical implications and in severe forms can preclude LT. After LT, the outcome is poor, with a mortality rate of 35% in patients with mean pulmonary artery pressure > 35mmHg. LT does not correct the situation.

### Renal problems after liver transplant

The true incidence of renal failure after LT is unknown due to the differences in the criteria and methods applied to evaluate renal function.^(31)^ It has been reported to vary from 5 to 50%, with 8 to 17% of recipients in need of renal replacement therapy. Approximately 10% of patients with renal dysfunction develop end-stage renal disease.^(5)^ Renal dysfunction (RD) can develop due to acute failure or exacerbation of an underlying preoperative dysfunction.^(31)^

There are several risk factors for acute RD, such as clinical status before transplant (MELD score, diabetes mellitus, hepatorenal syndrome, chronic renal disease), intraoperative events (hemodynamic disturbances, massive transfusion), several postoperative complications (infections, surgical re-exploration) and radiological investigations.^(31)^ Postoperative RD probably also occurs in the case of graft dysfunction, prolonged use of vasoactive agents, and drug-induced tubular injury (cyclosporine, tacrolimus, amphotericin, aminoglycosides).^(32)^

Renal complications generally occur in the early period following LT, and a main reason is misdistribution between fluid compartments and thus relative hypovolemia. Oliguria may be the first sign of RD; thus, close monitoring of hourly diuresis and ensuing correction of hypovolemia by adequate fluid replacement as well as avoidance of nephrotoxic drugs are needed.^(3,4)^

Use of terlipressin in preoperative HRS treatment has been shown to reduce postoperative RD development, and studies have shown that prophylactic use of fenoldopam provides perioperative renal vasodilation and decreases the risk of RD.^(31)^ Our group investigated this issue,^(33)^ but no specific therapy has been adopted.

Calcineurin inhibitor-induced RD is usually not observed in the early hours after LT, but in the presence of RD, drug doses should be adjusted and monitored through serial serum levels or removed from the immunosuppressive regimen. Calcineurin inhibitors injury is characterized by acute arteriopathy, although the true pathophysiology is not completely understood.^(33)^

If RD is serious enough to cause fluid retention, metabolic disturbances (acidosis, encephalopathy, etc.) and electrolyte imbalances should lead to renal replacement therapy (RRT). The criteria for RRT do not differ from the criteria generally used in critical care patients. Two issues must be taken into account: first, the usual biomarkers for RD may not be accurate in these patients; creatinine reflects muscular mass and function, and blood urea levels are closely related to liver function. Second, in oliguric patients, the fluid balance must be closely monitored to avoid fluid overload.^(34)^ This fluid overload can provoke peripheral and especially visceral edema, which contributes negatively to graft recovery.

Hepatic encephalopathy, deceased donor, MELD score, intraoperative blood loss and LT due to hepatocellular carcinoma have been identified as independent predictors for postoperative RRT.^(31)^

Basiliximab is a CD25 monoclonal antibody for the T-cell IL-2 receptor. It blocks T-cell activity by reducing their proliferation. Whenever renal comorbidity is seen after the LT basiliximab protocol for early-phase immunosuppression, the introduction of CNI therapy should be delayed.

### Neurological problems and management

Prior to transplantation, it is crucial to perform a baseline neuropsychiatric profile, and neurologic progression should be constantly checked, since neurological complications are very common after LT and prior symptoms increase the inherent risk. The most commonly observed neurological complications are encephalopathy, seizures and intracranial bleeding.^(35)^ Poor graft function may result in recurrence of encephalopathy, but its etiology is often difficult to determine accurately because multiple factors, such as subarachnoid hemorrhage, meningitis, infarction, spinal cord necrosis and cytomegalovirus infection, may be involved.^(35,36)^ Seizures are the second most common neurological complications, often preceded by some degree of encephalopathy, but may have other etiologies, such as stroke, metabolic disturbances, electrolyte disorders, drug toxicity, previous history of epileptic seizures and infection. Cerebral infarction may occur in the early perioperative period and is mostly the result of anoxic ischemic events, often preceded by hypotension.

Neurological manifestations connected to immunosuppression may establish following high-dose steroids and calcineurin inhibitors. They include headache, confusion/psychosis, lowering of seizure threshold, speech apraxia, myoclonus, visual hallucinations, tremor, delirium, cortical blindness and coma.^(35-37)^

Central pontine myelinolysis syndrome is one of the most severe neurological complications after LT and is characterized by symmetrical myelin loss at the pontine. There is no definitive treatment; however, it can be prevented through slow correction of hyponatremia and tight monitoring of serum sodium levels.^(37)^ Although also a feared complication, psychosis may exclusively result from a prolonged ICU stay, steroids, immunosuppression and drug interactions.^(37)^

Conditions that increase the risk of postoperative neurological impairment also include preexisting blood-brain barrier alterations causing toxic intracerebral drug levels, dysmetabolic alterations and electrolyte and osmotic disorders.^(35,36)^

### Infections early after liver transplant

The risk of infection early after liver transplantation is multifactorial and one of the major causes of postoperative morbimortality.^(38,39)^ Risk factors can be related to the donor, to the recipient and to transplantation *per se*. Transplant factors include IRI, intraoperative blood transfusion, level and type of immunosuppression, rejection, complications, prolonged intensive care stay, dialysis or ventilation supports, type of biliary drainage, repeated surgeries, retransplantation, antibiotics, antiviral regimen and environment. Donor risk factors include previous infection, prolonged intensive care unit stay, quality of the liver and viral status. For the recipient, the most important are MELD score above 30, other comorbidities before LT, malnutrition, RD, acute liver failure, previous infection or colonization and immune viral status (e.g., cytomegalovirus).^(38,39)^ The infectious agent can be bacterial, viral, parasitic or fungal.^(40)^ Risk factors for bacterial infections vary between transplantation centers and have different etiologies according to the time after transplantation.^(40)^ Prophylactic antibiotics are administered intraoperatively to decrease the risk of surgical site infections. More details about infections after LT are beyond our scope.

### Analgesia after liver transplant

Liver transplantation is among the major and most extensive abdominal surgeries, yet postoperative pain is not as severe as expected.^(41,42)^ Optimal pain control is essential in accelerating postoperative recovery; however, there is a wide variation in analgesic requirements among these patients, and titration of the analgesic effect to individual needs is the key to successful analgesia.

In LT patients, the reduced analgesic needs may be associated with the increased plasma levels of endogenous neuropeptides involved in pain modulation and with postoperative pharmacokinetic changes in the drugs that are typical in liver disease.^(43)^

Long-acting opioids are used with caution because they are associated with serious side effects and can precipitate subclinical encephalopathy.^(41)^ Fentanyl provides an acceptable degree of analgesia with minimal effects on liver function and hemodynamics.^(42)^ In many patients, combination treatment, for example magnesium metamizol or paracetamol in a low dose (< 2g a day) plus tramadol up to 300mg/day, is a reasonable and safe option in the immediate postoperative period, with analgesic superiority over single therapy and without additional toxicity.

Unrelieved pain may amplify the surgical stress response and organ dysfunction and prolong recovery.

### Monitoring graft function recovery

After multiple insults, the graft slowly recovers its function, reflected by an increase in factor V and INR normalization, usually observed in the first 24 hours after LT, occasionally up to 72 hours, after an initial graft dysfunction^(7)^ (Table 3). Factor V is synthesized only in the liver, is vitamin K dependent and has a short plasmatic half-life, making it a good marker monitoring liver function.^(44)^ Serial determination of serum lactate is also important, as it evaluates graft function recovery through hepatic lactate clearance and evaluates organ perfusion.^(45,46)^ Serum aminotransferases and bilirubin must be monitored; an unexpected increase in these parameters suggests complications, and vascular thrombosis must be immediately ruled out.^(47)^ Graft dysfunction influences biliary fluid, which becomes light colored and less viscous.

**Table 3:** Potential risk factors associated with liver graft dysfunction

Donor	Perioperative	Recipient
Age and sex	Warm ischemia	Age
Race	Technical complications	Comorbidities
Weight	Blood products use	Medical status
Cause of brain death		Renal insufficiency
ICU length of stay		Retransplantation
Cold preservation		Use of vasopressors
Serum sodium		
Use of vasopressors		
Steatosis		

ICU - intensive care unit.

The recovery time is related to several factors, such as ischemia time (cold and warm ischemia time), donor characteristics, and overall graft quality, namely, the presence of steatosis. Cold ischemia > 12 hours, warm ischemia > 1 hour and steatosis > 30% are considered risk factors for delayed recovery or early graft failure.^(3,4)^ Failure of the graft to recover adequate function defines primary nonfunction and indicates urgent retransplantation.

Routine Döppler ultrasonographic assessment of graft vascularization is important for the early detection of arterial or venous thrombosis.^(47)^ Early high arterial anastomotic resistance usually normalizes in a few days; if it persists along with hepatofugal flow in Döppler examination, it may reflect other graft problems, such as rejection.^(47,48)^

The green indocyanine test, although not used routinely in our center, can also evaluate graft function.^(47)^

Vascular thrombosis after LT may cause graft failure, immediate or long-term, and in most cases is an indication for urgent retransplant.^(48,49)^

Heparin is the anticoagulant of choice, with low-molecular-weight heparin (LMWH) preferred, because it is a selective inhibitor of coagulation factor Xa and has lesser hemorrhagic complications.^(21)^

Prophylactic doses of LMWH are usually started as soon as a normalization trend of TP, INR (< 1.5) and aPTT is registered and platelets are above 50.000 (in the absence of hemorrhagic diathesis) and are maintained for 2 weeks.^(48)^

Arterial thrombotic events, such as HAT, especially occur in the case of artery size discrepancy between the graft and native vessel, reduced arterial flow, increased sinusoidal resistance, preservation lesions or anastomotic stenosis.^(21,26,48)^ Studies focused on the efficacy and safety of antiplatelet therapy in patients after LT showed evidence of benefit either in early or late HAT without increasing the risk of bleeding or surgical complications.^(21,26)^ We currently use aspirin 100 mg per day introduced at D10 or earlier based on clinical judgment in patients at high risk for early HAT. Aspirin is kept up for 3 months or indefinitely if the risk for arterial thrombosis is maintained.

### Patients with familial amyloidotic polyneuropathy

In Portugal, which has a high incidence of familial amyloidotic polyneuropathy, liver transplantation is a possible therapy. Additionally, the FAP patients usually donate their livers to another patient in a domino surgical procedure.^(2,3)^ Several particular aspects must be taken into account. First, the liver is normal in function, except for the production of an abnormal protein. This factor protects these patients from major bleeding. Second, there is a higher incidence of HAT,^(50)^ so vascular monitoring is more frequent. In these patients, dysautonomic features are frequently present, and the response to vasopressors, especially inotropic agents, can be aberrant. One last issue is the possible recurrence of FAP disease in the recipient; this affects the patient selection and is not performed by intensive care physicians.

## CONCLUSION

We provide a standpoint for the approach to liver transplantation in our transplantation center, based on our experience. We discuss mainly the recipient's clinical profile, early complications, adequate approaches to them, and general management in the first posttransplantation hours.
